# The roles of essential trace elements in T cell biology

**DOI:** 10.1111/jcmm.18390

**Published:** 2024-05-27

**Authors:** Linbo Lan, Zhao Feng, Xiaobin Liu, Baojun Zhang

**Affiliations:** ^1^ Department of Medical Immunology, College of Basic Medical Sciences Yan'an University Yan'an China; ^2^ Clinical Teaching and Research Center, School of Nursing Weinan vocational and technical college Weinan China; ^3^ Department of Pathogenic Microbiology and Immunology, School of Basic Medical Sciences Xi'an Jiaotong University Xi'an Shaanxi China; ^4^ Xi'an Jiaotong University Health Science Center, Institute of Infection and Immunity, Translational Medicine Institute Xi'an Shaanxi China; ^5^ Key Laboratory of Environment and Genes Related to Diseases Xi'an Jiaotong University Xi'an Shaanxi China

**Keywords:** immune regulation, minerals, nutrients, T cells, trace elements

## Abstract

T cells are crucial for adaptive immunity to regulate proper immune response and immune homeostasis. T cell development occurs in the thymus and mainly differentiates into CD4^+^ and CD8^+^ T cell subsets. Upon stimulation, naive T cells differentiate into distinct CD4^+^ helper and CD8^+^ cytotoxic T cells, which mediate immunity homeostasis and defend against pathogens or tumours. Trace elements are minimal yet essential components of human body that cannot be overlooked, and they participate in enzyme activation, DNA synthesis, antioxidant defence, hormone production, etc. Moreover, trace elements are particularly involved in immune regulations. Here, we have summarized the roles of eight essential trace elements (iron, zinc, selenium, copper, iodine, chromium, molybdenum, cobalt) in T cell development, activation and differentiation, and immune response, which provides significant insights into developing novel approaches to modulate immunoregulation and immunotherapy.

## 1 INTRODUCTION

T lymphocytes (T cells) are essential components of the adaptive immune system that are crucial for maintaining human health.^1^ After the lymphoid precursor migrates from the bone marrow to the thymus, T cells differentiate into early T cell progenitors (ETPs), CD4^−^ CD8^−^ double‐negative (DN) cells. Next, ETPs undergo positive and negative selection and finally survive, resulting in mature CD4^+^ or CD8^+^ single‐positive (SP) T cells, named helper T cells (Th cells) and cytotoxic T cells (CTLs), respectively.^2^ Under the stimulation of TCR and costimulatory signals together with certain cytokines, Th cells can be differentiated into distinct subsets, including T helper 1 (Th1), T helper 2 (Th2), T helper 17 (Th17), T helper 9 (Th9), T follicular helper cell (Tfh), and regulatory T cells (Tregs). They can either boost or regulate immune responses through supporting antigen‐presenting cells (APCs), CD8^+^ T cells, B cells, etc.^3^ In contrast, CD8^+^ T cells differentiate into CTLs after recognizing antigens presented by APCs and then defend against pathogens or tumours.^4^

Trace elements are primarily inorganic salts, also referred to as minerals. Their existence in the human body is minimal, accounting for less than 0.01% of total body mass. According to World Health Organization (WHO) standards, the human body contains eight essential trace elements: iron (Fe), zinc (Zn), selenium (Se), copper (Cu), iodine (I), chromium (Cr), cobalt (Co) and molybdenum (Mo).^5^ In addition to physiological processes, such as enzyme function, hormone production, DNA synthesis, and antioxidant defence, these essential trace elements are also involved in the development and function of the immune system.^6^ Recently, increasing studies have found that proper T cell immunity depends on the adequate action of essential trace elements. This review will focus on summarizing the role of essential trace elements in T cell development, activation, differentiation, and immune response (Table 1), which might provide valuable insights into immunoregulation and immunotherapy.

**TABLE 1:** Effects of essential trace elements on T cells.

Trace elements	Element state	Impact on T cells	Disease‐related outcomes	Potential applications
Iron	Iron supplementation	T cell activation↑^7^	Iron deficiency: IDA (Th1↓)^8^; Iron overload: Salmonella enterica serovar infection accompanied (Th1↓)^9^	Exogenous supplementation: SLE (Tfh↑)^10^; Endogenous release: Cryo‐thermal therapy for tumours (CD4^+^CTL↑, Tfh↑, Th2↓, Th17↓)^11^
	Iron deficiency	T cell development: thymocyte proliferation↓, thymic atrophy, DN stage blockade^12^, ^13^; T cell differentiation: Th1↓^8^		
	Iron overload	T cell development: disruption T cell DNA synthesis and cell cycle entry^14^; T cell differentiation: Th1↓, Tfh↑^9^, ^15^		
Zinc	Zinc supplementation	T cell activation↑^16^, ^17^; T cell differentiation: CD4^+^ T↑, CTL↑, Th1↑, Treg↑, Th9↓, Th17↓^18^, ^19^, ^20^, ^21^, ^22^	Zinc deficiency: Asthma (Th2↑)^23^; Chronic sinusitis (Th2↑)^24^; Colonic inflammation (Th17↑)^25^	Exogenous supplementation: AIDS (normalization of T cell production and reduction of infection)^26^; Sickle cell disease (TNF‐α↓, IL‐1β↓)^27^; EAE (Th17↓)^28^; GVHD(Treg↑)^21^
	Zinc deficiency	T cell development: thymosin activity↓, thymic epithelial cell↓, thymocytes apoptosis↑, and pre‐T cell apoptosis↑^29^, ^30^, ^31^; T cell differentiation: Th2↑, Th17↑^25^, ^32^		
	Zinc overload	T cell activation↓^33^		
Selenium	Selenium supplementation	T cell activation↑^34^, ^35^, ^36^; T cell differentiation: Th1↑, Treg↑^37^, ^38^	Selenium deficiency: Tuberculosis accompanied by selenium deficiency (T cell activation↓)^39^	Exogenous supplementation: AIDS (CD4^+^T activation↑, proliferation↑, CD4^+^T exhaustion↓)^40^; Breast cancer model (Th1↑)^37^
	Selenium deficiency	T cell activation↓^39^; T cell differentiation: Th1↓, Th2↑^41^, ^42^		
Copper	Copper deficiency	T cell activation↓ and proliferation↓^43^	Copper overload: Copper ions overload Mice chondritis (CD4^+^ T cell↑)^44^; Inhaling CuONPs (induction of pulmonary inflammation and IFN‐γ↑, IL‐4↑, IL‐5↑)^45^, ^46^	Exogenous inhibition: Copper‐lowering therapy Tumour (CD4^+^ T cells exert anti‐tumour into tumour tissues↑)^47^
	Copper overload	T cell differentiation: CD4^+^T cell↑^44^		
Iodine	Iodine supplementation	T cell differentiation: Treg↑, CTLs↑^48^	Iodine overload: Autoimmune thyroiditis (Th17 infiltration↑, thyroid cells apoptosis↑, and thyroid cells↓)^49^; AIT mice (Treg↓)^50^	Radiation therapy: Treating thyroid cancer with Iodine‐131 (Th17↓, Treg↓)^51^; Treating prostate cancer with iodine‐125 (CD4^+^T↑, CD4/CD8↑)^52^
	Iodine overload	T cell differentiation: Treg↓, Th17 infiltration↑^49^, ^50^		
Molybdenum	Molybdenum deficiency	T cell development: DN proportion↓, thymus atrophy^53^	NA	NA
Cobalt	Cobalt overload	T cell numbers↓^54^	NA	Arthroplasty of the hip with cobalt graft (T cell activation↑, pseudotumor contribution)^55^
Chromium	Chromium (III) overload	T cell proliferation↑^56^	Chromium(VI) overload: Exposed to chromium (VI) and resulting in incidence of nasal injury (T cell numbers↓, IFN‐γ↓, IL‐6↓, IL‐10↓, and IL‐17A↓)^57^	NA
	Chromium (VI) overload	T cell development: thymocyte apoptosis↑, thymocytes↓^58^		

Abbreviations: AIDS, acquired immunodeficiency syndrome; AIT, autoimmune thyroiditis; CuO NPs, copper oxide nanoparticles; EAE, autoimmune encephalomyelitis; GVHD, graft‐versus‐host disease; IDA, iron deficiency anaemia; SLE, systemic lupus erythematosus.

## 2 THE ROLE OF ESSENTIAL TRACE ELEMENTS

### 2.1 Iron

Iron is the most abundant essential trace element, weighing 4–5 g, with 60%–70% bound to haemoglobin, which transports oxygen and carbon dioxide in the body.^59^ The iron‐importing protein transferrin (Tf) is responsible for transporting and distributing almost all iron within circulating blood.^60^ Iron translocation from Tf to cells predominantly occurs through endocytosis mediated by the transferrin receptor (TfR) complex.^61^ Iron is a catalyst for enzymes and is critical in DNA synthesis and repairing cellular energy metabolism.^62^ However, iron overload can lead to increased production of hydroxyl radicals through the Fenton reaction, resulting in oxidative stress and damage to cells and tissues.^63^ Iron participates in the development and activation of T cells, and alterations in iron concentrations modulate T cell differentiation and function during the immune responses.

In the context of T cell development, iron deficiency reduces thymocyte proliferation and maturation, leading to thymic atrophy.^12^ Likewise, when the TfR is damaged or inhibited, T cells cannot capture the iron required for development and are blocked at the CD4^−^ CD8^−^ DN stage.^13^ Iron overload decreases TfR expression, which impedes T cell development by disrupting DNA synthesis and cell cycle entry.^14^

The iron has also been shown to influence the differentiation of mature T cells. First, iron is a requirement to promote T cell activation through TfR via an IL‐2‐dependent pathway.^7^ Iron deficiency reduces the Th1 cells subpopulation, which has also been observed in paediatric patients diagnosed with iron deficiency anaemia (IDA).^8^ The study indicates that iron chelation decreases IL‐12 levels, thereby inhibiting Th1 differentiation and reducing IFN‐γ secretion.^64^ Notably, timely administration of iron effectively reverses this abnormality.^65^ In chronic infection models with Salmonella enterica serovar typhimurium, iron overload reduces Th1 differentiation. This is mainly related to iron upregulate T cell mucin‐containing protein‐3 (TIM‐3) expression.^9^ The Cryo‐Thermal Therapy (a therapeutic approach for the management of cancer) could result in the release of large amounts of iron in the tumour area, which promotes CD4^+^ T cell differentiation into CTL and Tfh cells, inhibits CD4^+^ T cell differentiation into Th2 and Th17 cells, and effectively digests tumours.^11^ In several diseases, changes of iron concentration can promote Treg cell differentiation, thereby suppressing immune responses. In thalassemia patients, there is a positive correlation between Tregs and ferritin concentration,^66^ and iron overload is more susceptible to infection with Yersinia spp, Listeria monocytogenes, and Vibrio vulnificu.^67^ We suspect that the increased risk of infection may be related to the immunosuppressive response of Tregs. Similarly, Treg proliferation also occurs in myelodysplastic patients with iron removal therapy, which reduces the negative effects of T‐cell‐mediated immune damage on haematopoiesis.^68^ Moreover, iron is regulated by miR‐21/BDH2 axis in systemic lupus erythematosus, gradually accumulates and leads to more pronounced differentiation of Tfh cells, aggravates the disease severity, and iron removal reduces the proliferation of pathogenic Tfh cells and alleviates disease progression.^10^

Recently, ferroptosis is identified as a form of regulatory cell death closely associated with iron metabolism, and high levels of iron can promote ferroptosis by inducing oxidative stress and lipid peroxide.^69^ In a mouse lymphocytic choriomeningitis virus infection model, the inactivation of mTORC2‐AKT‐GSK3β axis and GPX4 peroxidase activity would trigger virus‐specific memory CD4^+^ T cell ferroptosis.^15^ The mechanism differs from memory CD4^+^ T cells, NIX defects in memory CD8^+^ T cells will increase mRNA expression of PTGS2 (a marker gene associated with ferroptosis), which may be associated with the reduced survival of memory CD8^+^ T cells.^70^

### 2.2 Zinc

Zinc is the second most abundant essential trace element after iron, with a standard zinc content in the human body of 1.4–2.3 g. Zinc is a pivotal component of proteins, including enzymes and transcription factors, which regulate DNA replication, gene transcription and signal transduction.^71^ Zinc also controls immunological processes, such as maintaining the integrity of the tissue barriers, preventing pathogen infection,^72^ and regulating T cell development, activation, differentiation, and functions.

First, zinc is involved in thymus size and thymocyte development. On one side, zinc deficiency causes thymosin activity to decrease, leading to lower thymic epithelial cell development and maturity.^73^ On the other side, zinc deficiency increases the expression of the protein p56^lck^, potentially heightening the susceptibility of thymocytes to apoptosis.^29^, ^30^ In addition, zinc deficiency leads to an enhancement of apoptosis in pre‐T cell progenitors via altering the Bcl‐2/Bax pathway, and zinc supplementation reverses this change by inhibiting caspase‐3, −6, −7 and −8.^31^ In acquired immunodeficiency syndrome (AIDS) patients treated with zinc, T cell production returned to normal percentages and effectively reduced the infections.^26^

Second, zinc profoundly impacts T cell activation and function. Zinc promotes T cell activation through the synthesis of protein kinase C and lymphocyte protein tyrosine kinase (LCK).^16^, ^17^ However, T cell activation can be inhibited in the presence of a zinc overdose in culture.^33^ Besides, zinc reduces the incidence of infection by increasing the number of CD4^+^ T cells and CTL, mainly by increasing the production of IL‐2 and sIL‐2R.^18^

Third, zinc broadly regulates Th cell differentiation and immune balance. Zinc supplementation induces a notable alteration in the balance between Th1 and Th2 cells, favouring the Th1 immune response. This is mainly due to up‐regulated interferon‐γ (IFN‐γ) and transcription factor T‐bet expression.^19^ Conversely, zinc deficiency promotes Th1 cells towards Th2 cell differentiation. Alongside, there is a reduction in the synthesis of Th1 cell cytokines, including IFN‐γ, interleukin‐2 (IL‐2), and tumour necrosis factor‐alpha (TNF‐α), an increase in the production of Th2 cytokines, such as interleukin‐4 (IL‐4), interleukin‐6 (IL‐6) and interleukin‐10 (IL‐10).^32^ The increase of Th2 cells exacerbates pain and organ dysfunction in patients with sickle cell disease and causes adverse immune responses to diseases such as asthma or chronic sinusitis.^23^, ^24^ Oral zinc acetate protects patients with sickle cell disease from upper respiratory tract infection, reducing the proinflammatory cytokines TNF‐α and IL‐1β secreted by Th1 cells.^27^

Zinc hinders Th17 cells differentiation via impeding the IL‐6/STAT3 signalling pathway or IL‐1 receptor‐associated kinase 4 (IRAK4) phosphorylation.^20^ Similar clinical data reveal that zinc is involved in autoimmune diseases, such as autoimmune encephalomyelitis.^28^ Zinc deficiency activates the IL‐23/Th17 axis to aggravate colonic inflammation with increased Th17 cells in mice.^25^ Moreover, zinc supplementation suppresses sirt‐1 deacetylase activity, resulting in the induction of forkhead box P3 (Foxp3) expression, which facilitates Treg differentiation and contributes to the prevention of graft‐versus‐host disease (GVHD).^21^ In addition, zinc downregulates STAT6 phosphorylation which inhibits Th9 differentiation and reduces IL‐9 secretion, thereby dampening allogeneic immune reaction.^22^

### 2.3 Selenium

The total amount of selenium is 3–20 mg in the human body. The biological functions of selenium are mainly mediated through selenoproteins in organisms.^74^ For example, selenium synthesizes glutathione peroxidase (GPx), which acts as an antioxidant, helping to scavenge free radicals and protect cell membranes.^75^ Selenium synthesizes thioredoxin reductase (TxnRd) to maintain intracellular redox status and cell viability.^76^, ^77^ Moreover, selenium is involved in overall process of immune responses, such as T cells activation, proliferation and differentiation.

Selenium can promote T cells activation by various mechanisms. First, selenium enhances the expression of the alpha (p55) and/or beta (p70/75) subunits of the IL‐2R, facilitates their interaction with IL‐2, and increases T cell activation and proliferation,^34^, ^35^ which has been demonstrated in clinical trials. For instance, oral selenium improves immune response through an earlier peak of T cell proliferation.^78^ In AIDS patients, selenium can promote the activation and proliferation of CD4^+^ T cells, reduce the exhaustion of CD4^+^ T cells, and decrease HIV viral load.^40^ In addition, selenium inhibits ROS production and thus promotes T cell activation.^36^ In tuberculosis patients, selenium deficiency leads to elevated intracellular ROS and oxidative stress in T cells and reduces T cell activation; timely selenium supplementation can restore GPx function to relieve symptoms.^39^

Selenium levels also regulate the differentiation of Th cell subsets. As shown in mouse breast cancer model, selenium supplementation can induce Th1 differentiation and secrete more IFN‐γ to reduce tumour volume in favour of the prognosis.^37^ However, selenium deficiency leads to Gpx deficiency in vivo, therefore decreases the Th1 differentiation and promotes Th2 differentiation.^41^, ^42^ Treg cells were increased with increasing dietary selenium, which may be related to the decrease levels of nuclear factor‐κB ligand.^38^ Selenium‐synthesized GPX4 has also been shown to protect Tfh cells from ferroptosis, thereby enhancing germinal center responses.^79^

### 2.4 Copper

The copper content in the human body is 100–150 mg. Dietary copper is absorbed by the intestinal epithelial cells and transported to the liver through the portal vein. Copper binds with albumin and ceruloplasmin in the liver and is carried throughout the body via the bloodstream.^80^ Copper is vital for maintaining essential cellular functions, as well as involving antioxidant reactions, maintaining cell membrane and DNA integrity, and aiding in ATP production.^81^

Copper deficiency leads to reduced IL‐2, which inhibits T cell activation and proliferation,^43^ and copper supplementation reverses this change.^82^ Copper ions can induce auricular chondritis in mice, which causes CD4^+^ T cell proliferation and promotes the release of IFN‐γ, IL‐2, and TNF‐α from Th1 cells.^44^

In recent decades, the production and use of copper oxide nanoparticles (CuO NPs) have expanded. As a result, these particles enter the body through the respiratory tract, causing high levels of copper in the lungs and liver.^45^ Inhaling CuO NPs promotes Th1 cells to secrete IFN‐γ, and Th2 cells to secrete IL‐4 and IL‐5, leading to pulmonary inflammation.^45^, ^46^

Copper can also regulate T cell‐mediated antitumor immunity. Copper up‐regulates the expression of PD‐L1 by stimulating the IL6/JAK/STAT3 signalling pathway, leading to the exhaustion of T cells and the inability to kill tumour cells.^83^ In neuroblastoma cell assays, copper chelators inhibit STAT3 phosphorylation, promote ubiquitin‐mediated degradation of PD‐L1, increase the number of infiltrated CD8^+^ T cells and slow tumour growth.^84^ In mesothelioma mice treated with copper‐lowering therapy, CD4^+^ T cells may exert anti‐tumour immunity through the specific adhesion molecule CD40 into tumour tissues.^47^ Apparently, using copper‐lowering as an antitumor strategy provides new ideas for tumour therapy.

Last, copper accumulation triggers aggregation of lipoylated proteins, leading to loss of iron–sulfur cluster proteins. This process disrupts the tricarboxylic acid cycle pathway, inducing proteotoxic stress and ultimately causing cell death, a phenomenon termed cuproptosis.^85^ In the context of osteosarcoma, cuproptosis‐related lncRNAs may diminish Treg cell populations, enhancing the efficacy of anti‐PD‐1 antibodies.^86^ In addition, cuproptosis‐related genes (CRGs) may influence T cell exhaustion phenotype and serve as effective prognostic indicators for lung adenocarcinoma.^87^ In Temporal lobe epilepsy, upregulated CRGs may promote peripheral CD4^+^ and CD8^+^ T cell infiltration into epileptic focus, which increases seizure frequency.^88^ In patients with pancreatic adenocarcinoma, CRGs may drive the infiltration of antitumor components, such as activated CD4^+^ and CD8^+^ T cells, which are associated with longer survival.^89^ Further, copper‐coated nanomaterial sticking to T cell membrane could target the triple‐negative tumour cells to promote cuproptosis, offering promising applications in tumour immunotherapy.^90^

### 2.5 Iodine

The human body contains 20–50 mg of iodine, and iodine increases antioxidant activity, stabilizes the cellular redox state and inactivates pro‐inflammatory pathways.^91^, ^92^ It is widely believed that iodine is required to maintain normal organ development and function by synthesizing thyroxine.^93^

Iodine can directly affect the differentiation of multiple T cell subsets and regulate the corresponding immune responses. In response to intracellular antigens, iodine activates the Th1 response, increases IL‐2 and IFN‐γ production, promoting effector CTLs activation and proliferation. In response to extracellular antigens, iodine activates the Th2 response, increases cytokine secretion (IL‐6, IL‐10, IL‐8), and promotes Treg activation and differentiation.^48^ The exact mechanism of this differential response is unknown, but it has been predicted to be related to epigenetic modifications by iodine‐mediated activation of demethylase (DMT3).^94^ High‐dose iodine intake increases the incidence of autoimmune thyroiditis (AIT), leading to infiltration of Th17 cells in the thyroid gland, as well as triggering aberrant expression of TNF‐associated apoptosis‐inducing ligand (TRAIL) in thyroid cells, which induces apoptosis and parenchymal destruction of thyroid cells.^49^ In iodine induced AIT mice, oral overdosed iodine reduces Foxp3 mRNA expression and decreases Treg cells in spleen, which will aggravate gradually with the extension of iodine intake.^50^ Histologic analysis of the thyroid gland showed that following iodine therapy in AIT, the expression of MHC class I molecules on thyroid follicular cells was elevated, and CD8^+^ T cells began to infiltrate the gland.^95^

Radioactive iodine is also involved in regulating T cells in the context of tumours. Patients with differentiated thyroid cancer (DTC), higher levels of Th17 and Treg cells, and serum IL‐17, IL‐23, IL‐10, and TGF‐β1 were observed compared to healthy individuals. However, after treatment with radioactive iodine 131, this change has been reversed, which contributes to the recovery of DTC patients.^51^ In treating prostate cancer, iodine‐125 (I‐125) therapy increases the percentage of CD4^+^ T cells and the ratio of CD4/CD8. It also decreases peripheral serum prostate‐specific antigen, which indicates it has suppressed prostate cancer.^52^ On one hand, CD4^+^ T cells can differentiate into Th cells, which help to enhance other immune cells killing the tumour, and on the other hand, CD4^+^ T cells can differentiate into CD4^+^ CTL cells directly killing the tumour. The antitumor effect of I‐125 may be related to the increased CD4^+^ T cells, which needed to be clarified.

### 2.6 Other essential trace elements

#### 2.6.1 Chromium

The human body contains 5–10 mg of chromium, and chromium usually exists in the form of trivalent chromium (III) and hexavalent chromium (VI).^96^ Chromium (III) salts, such as chromium poly nicotinic acid, chromium chloride, and chromium picolinate, have been used as nutritional supplements to affect the metabolism of sugars, proteins, and lipids.^97^ Appropriate amounts of chromium salts enhance T cell proliferation with the stimulation of mitogen concanavalin A (Con‐A) and phytohemagglutinin.^56^ However, chromium (VI) is a toxic substance that induces apoptosis of thymocytes, leading to a reduction in thymocytes and thymic mass.^58^ Exposure to chromate (VI) leads to a reduction of T cell numbers, and the expression of IFN‐γ, IL‐6, IL‐10 and IL‐17A.^57^ Continuing exposure further results in the immunosuppression and inflammation, as well as a higher incidence of nasal injury.^98^

#### 2.6.2 Molybdenum

Molybdenum levels are approximately 9 mg in the body, which acts as a cofactor to catalyse redox reactions throughout the carbon, nitrogen, and sulfur cycles.^99^, ^100^ Molybdenum deficiency can decrease DN proportion and cause significant thymus atrophy by inhibiting ribosomal protein expression.^53^ And in ducks, molybdenum deposition disrupts the balance between Th1 and Th2 cells, and an increase TNF‐α, IFN‐γ, IL‐6, and IL‐8 secretion, which leads to endoplasmic reticulum stress and splenocyte apoptosis.^101^

#### 2.6.3 Cobalt

The cobalt content in the human body is nearly 1.0 mg, which accelerates red blood cell regeneration and haemoglobin synthesis.^102^ Cobalt is poisonous to T cells and decreases the total circulating T cell population.^54^ The insertion of a cobalt graft may lead to T cell activation, but these T cells are anergic and sensitized, which contributes to the formation of pseudotumors.^55^ Clinical reports have shown that CoCrMo alloy grafts activate T cells in patients. In female patients, T cells produce more IL‐10 and increase osteoclast formation and function. This may reduce bone remodelling around the implant osseointegration surface, resulting in implant loosening.^103^

## 3 CONCLUSION

**FIGURE 1:**
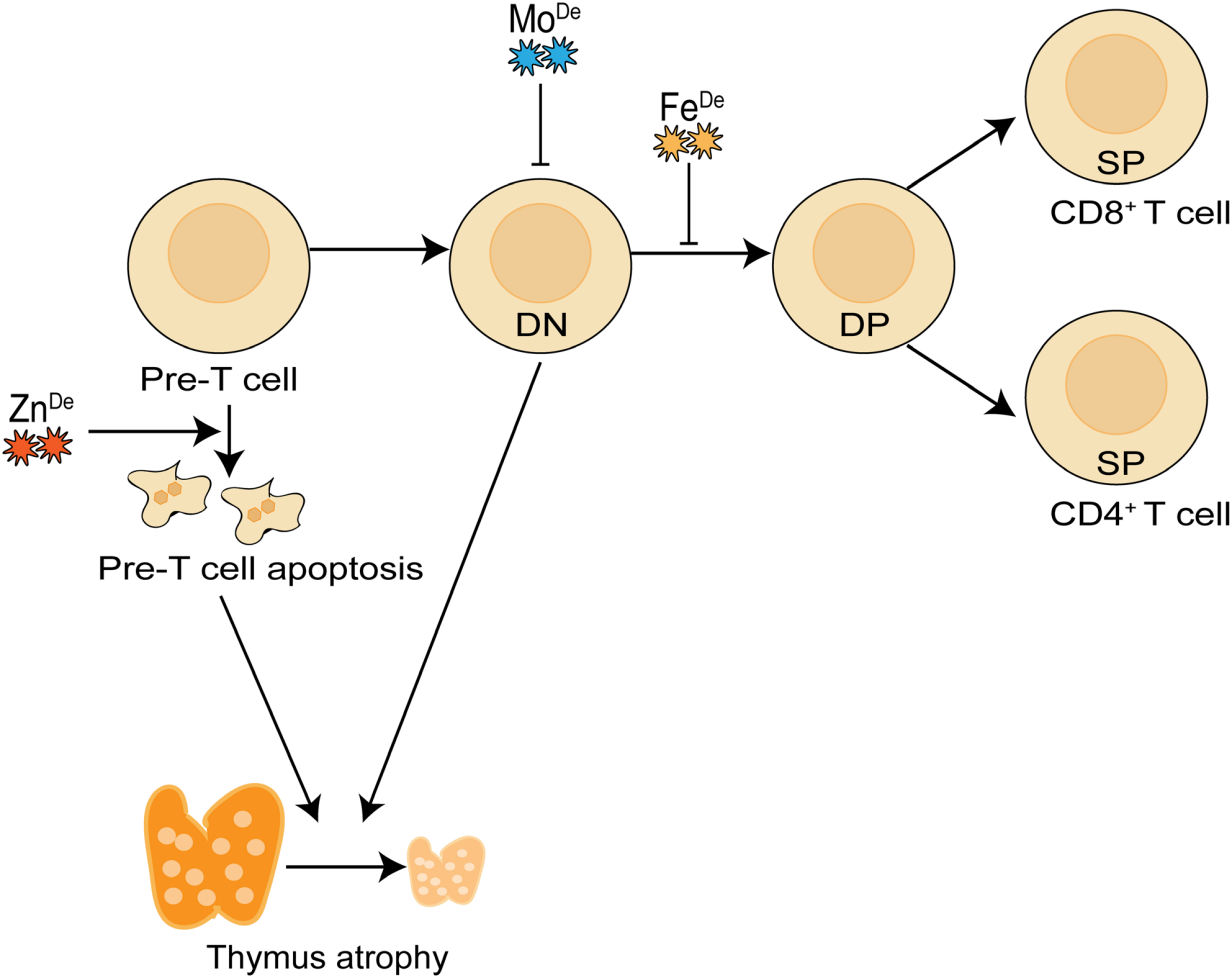
The effects of trace elements on T cell development. Zinc deficiency leads to apoptosis of pre‐T cells, iron deficiency blocks T cell development at the CD4^−^ CD8^−^ DN stage, and a deficiency in molybdenum results in a decrease in DN cells, ultimately causing thymic atrophy. ‘De’ represents for deficiency.

**FIGURE 2:**
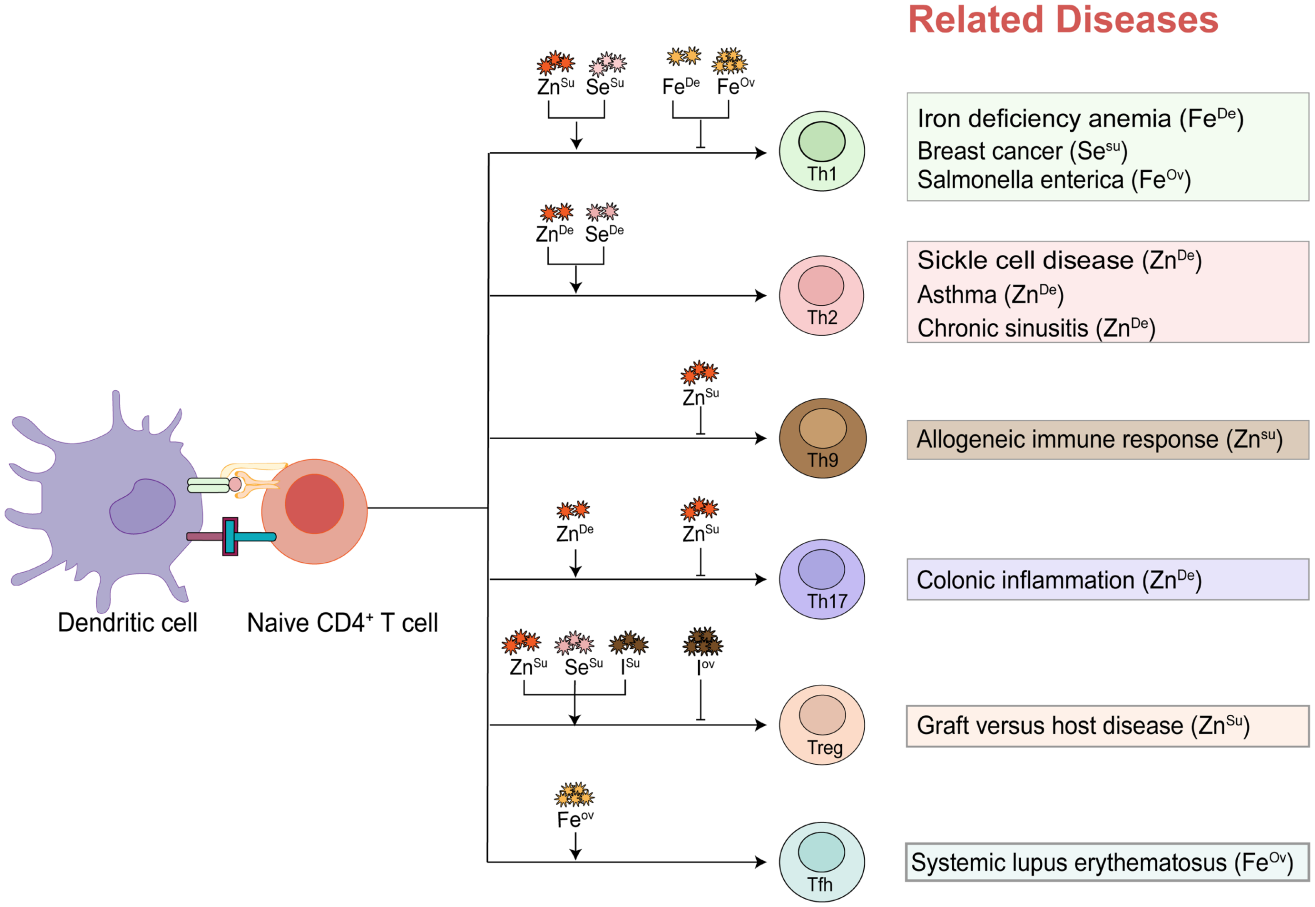
The impact of essential trace elements on T cell differentiation. The differentiation of T cells towards Th1 is reduced under conditions of iron deficiency or iron overload, a finding confirmed in patients with IDA and Salmonella enterica infections. Supplementation with zinc and selenium can promote Th1 differentiation, where selenium supplementation can increase Th1 cells, reducing the volume of breast tumours and exerting an anti‐tumour effect. Deficiencies in both zinc and selenium stimulate Th2 differentiation, with zinc deficiency leading to a shift towards Th2, exacerbating pain and organ dysfunction in patients with sickle cell disease, and causing adverse immune responses to diseases such as asthma or chronic sinusitis. Zinc supplements inhibit Th9 differentiation, dampening the allogeneic immune reaction. Zinc deficiency promotes Th17 differentiation, worsening colonic inflammation, while zinc supplementation can inhibit its differentiation. Supplementing with zinc, selenium, and iodine enhances Treg cell differentiation, however, overdosed iodine can reduce Treg cell differentiation. Zinc supplementation can promote Treg differentiation and prevent the occurrence of GVHD. Iron overload promotes Tfh differentiation and exacerbates symptoms in SLE patients. ‘Ov’ represents for overload; ‘Su’ represents for supplementation; ‘De’ represents for deficiency.

Essential trace elements are key contributors to an optimal function of T cell immunity. The present review has summarized the roles of essential trace elements in regulating T cell development (Figure 1), activation, differentiation (Figure 2), and immune response. Better understanding the key roles of essential trace elements involved in T cell immunity might accelerate the application of their supplement in immune regulation as well as minimize side effects. Of note, further exploring the roles of essential trace elements in regulating the development, differentiation, and function of T cells as well as other immune cells, will provide novel perspectives and strategies for T cell‐based immunotherapies.

## AUTHOR CONTRIBUTIONS

**Linbo Lan:** Resources (equal); writing – original draft (equal); writing – review and editing (equal). **Zhao Feng:** Resources (equal); writing – original draft (equal); writing – review and editing (equal). **Xiaobin Liu:** Conceptualization (equal); writing – review and editing (equal). **Baojun Zhang:** Conceptualization (equal); funding acquisition (equal); writing – review and editing (equal).

## FUNDING INFORMATION

This work was supported by grants from the National Key Research and Development Program of China (201YFA1100702, to B.Z.); grant from the National Natural Science Foundation of China (32170892, to B.Z.); Key Research and Development Program of Shaanxi Province (2022GXLH‐01‐16, to B.Z.); and Fundamental Research Funds for the Central Universities (xtr072022002, to B.Z.).

## CONFLICT OF INTEREST STATEMENT

The authors confirm that there are no conflicts of interest.

## PERSPECTIVES

The regulation of trace elements in T cells has shown therapeutic effects across various diseases. Numerous clinical studies have substantiated the pivotal role of trace elements in modulating T cell responses and their associations with diseases. Specifically, zinc and selenium supplementation have been shown to bolster the proportion of T cells in AIDS patients. Moreover, zinc supplementation can reduce the incidence of infection by increasing the number of CD4^+^ T cells and CTLs. Selenium supplementation can induce Th1 cells to secrete IFN‐γ, which correlates with a decrease in breast cancer volume. Reduced iron levels in patients with SLE have been found to decrease the number of pathogenic Tfh cells, and thereby ameliorate symptoms. Additionally, lowering copper levels in tumour tissues has been shown to enhance the ability of CD4^+^ T cells to target and eradicate tumours. These investigations provide potential applications of trace elements in clinics through regulating T cell immunity.

## Data Availability

The data that support the findings of this study are available from the corresponding author, [Baojun Zhang], upon reasonable request.
